# Establishment and Performance Evaluation of Multiplex PCR-Dipstick DNA Chromatography for *Mycoplasma pneumoniae* and *Chlamydia pneumoniae* Rapid Detection

**DOI:** 10.1155/2023/6654504

**Published:** 2023-09-28

**Authors:** Liuyang Hu, Xiuri Wang, Donglin Cao, Qiuchen Cheng, Qiong Li

**Affiliations:** ^1^Department of Laboratory Medicine, The People's Hospital of Guangxi Zhuang Autonomous Region, Guangxi Academy of Medical Sciences, Nanning 530016, China; ^2^Department of Gastroenterology, The People's Hospital of Guangxi Zhuang Autonomous Region, Guangxi Academy of Medical Sciences, Nanning 530016, China; ^3^Department of Laboratory Medicine, Guangdong Second Provincial General Hospital, Guangzhou 510317, China; ^4^Guangzhou Biotron Technology Co., Ltd., Guangzhou 510336, China

## Abstract

**Methods:**

Nasopharyngeal swab samples of 300 children with an acute respiratory tract infection were detected by a multiplex PCR-dipstick chromatography assay, and the results were compared with the DNA sequencing and serum IgM antibody assay.

**Results:**

A multiplex PCR-dipstick DNA assay can specifically detect *Mycoplasma pneumoniae* and *Chlamydia pneumoniae* and shows a good specificity, with a minimum detection limit of 10 CFU/mL, respectively. Using DNA sequencing results as the gold standard, the sensitivity, specificity, positive predictive value, and negative predictive value of the multiplex PCR-dipstick DNA chromatography assay for the diagnosis of *Mycoplasma pneumoniae* were 96.61%, 100%, 100%, and 99.18% respectively, and those of *Chlamydia pneumoniae* were 95.24%, 100%, 100%, and 99.64% respectively. There was no statistical significance MP and CP diagnosis by the multiplex PCR-dipstick DNA assay and DNA sequencing (MP: *P* = 0.5; CP: *P* = 1.0), and the two assays had very high statistical consistency (MP: kappa = 0.979; CP: kappa = 0.974). The positive rate of the multiplex PCR-dipstick chromatography assay was significantly higher than that of the serum IgM antibody assay, with MP (17.7% vs. 13.3%), CP (5.7% vs. 3.3%), and mixed infection of MP and CP (1.3% vs. 0.67%).

**Conclusions:**

A multiplex PCR-dipstick chromatography assay was successfully established for the joint detection of *Mycoplasma pneumoniae* and *Chlamydia pneumoniae* within 2 hours. It is simple, fast, sensitive, accurate, cost-effective with good diagnostic performance, which can be used for small laboratories and point-of-care diagnosis.

## 1. Introduction

Acute respiratory tract infections (ARTIs) are one of the main causes of hospitalization and death in children under 5 years old [1]. At present, respiratory viruses are still the most common pathogens [2], but *Mycoplasma pneumoniae(M. pneumoniae*, MP) and *Chlamydia pneumoniae* (*C. pneumoniae*, CP) infections are becoming increasingly prominent in respiratory infections, causing approximately 10–40% and 5–10% of children's infections, respectively, and there are 1-2% mixed infections of *M. pneumoniae* and *C. pneumoniae* [3–5]. The clinical symptoms of infection with *M. pneumoniae* and *C. pneumoniae* lack specificity, and empirical treatment with macrolide antibiotics may lead to an increase in the drug resistance rate. Currently, the drug resistance rate of *M. pneumoniae* ranges from 85% to 100% [6–8]. Therefore, it is important to have a rapid and accurate laboratory diagnosis before conducting antibacterial treatment. The widely used identification methods in clinical include isolating culture, serum antibody testing, and molecular biology. *M. pneumoniae* rapidly proliferates in the culture medium, decomposes glucose to produce acid, and changes the pH value of the culture medium so that *M. pneumoniae* can determine the culture positive by observing the color change of the culture medium. However, M. pneumoniae cultivation conditions are harsh, time-consuming, and the positive rate is low [9, 10], which cannot meet the needs of rapid clinical diagnosis. *C. pneumoniae* is a microorganism that grows within cells and cannot be cultured in vitro. For the serum antibody detection method, MP-IgM and CP-IgM increase around 1 week of onset, reaching a peak at 3–6 weeks [11]. In the acute phase (within 1 week of onset), MP-IgM is found to be positive in 14%–45% patients [12], so this method cannot achieve the detection purpose in the early stage of infection. The exact sensitivity and specificity of PCR methods vary with the corresponding amplification platform and detection form [13]. Routine PCR needs to be analyzed in agarose gel electrophoresis, which takes a long time and has low sensitivity. The real-time fluorescent PCR method has high sensitivity and good detection specificity, but the requirements for complex equipment and well-trained personnel make it difficult to be widely used in small community hospitals and point-of-care diagnosis [14]. Loop-mediated isothermal amplification (LAMP) technology is a relatively new form of nucleic acid amplification testing, widely used for direct detection of clinically important pathogens, including *M. pneumoniae* [15]. However, traditionally, detecting turbidity changes in reaction mixtures requires the use of a spectrophotometer, which will limit its widespread use in restricted environments. Although visual detection reagents (VDRs) are an alternative method, the results may become less accurate at low template concentrations [16]. PCR-based DNA biosensors have been designed for the detection of pathogens and drug-resistant genes in the form of test strip nucleic acid detection, with advantages such as rapidity, specificity, sensitivity, low detection limit, and cost-effectiveness [17–21]. This study aimed to establish a multiplex PCR-dipstick DNA chromatography assay for the joint detection of *M. pneumoniae* and *C. pneumoniae* based on PCR and strip DNA biosensors.

## 2. Materials and Methods

### 2.1. Standard Strain

Standard strains of *M. pneumoniae* (ATCC15531) and *C. pneumoniae* (ATCC53592) were purchased from the Guangdong Culture Collection Center.

### 2.2. Clinical Samples

The study was approved by the medical ethics committee, and consent was obtained from patients' parents or legal guardians. 300 nasopharyngeal swab specimens and serum samples from hospitalized children with acute respiratory infections at the Second People's Hospital of Guangdong Province and the People's Hospital of Guangxi Zhuang Autonomous Region from January 2021 to December 2022 were collected. Nasopharyngeal swab specimens were detected by the multiplex PCR-dipstick DNA chromatography assay, and all PCR products were sent to DNA sequencing. Serum was used for MP-IgM and CP-IgM detection, which MP-IgM and CP-IgM positive indicates corresponding infection.

### 2.3. Sample Preparation

The nasopharyngeal swab sample was placed in 1 mL sterile physiological saline, stirred 20 times, and centrifuged at a speed of 15,000 rpm for 5 minutes, and then, the supernatant was discarded as much as possible. According to the manufacturer's instructions, the Magen HiPure bacterial DNA kit was used to extract genomic DNA content from nasopharyngeal swabs. The serum samples were centrifuged at a speed of 3,000 rpm for 5 minutes and sent to the laboratory for MP-IgM and CP-IgM testing.

### 2.4. Primer and Probe Design

Primers were designed according to the conserved sequence of MP and CP using prime 5.0. The 5′ end of the forward primer is labeled with oligonucleotide (Tag), the 5′ end of the reverse primer is labeled with biotin, and the forward primer end is connected to Tag with spacer C3, which can ensure that Tag remains single-stranded during amplification. In order to verify the DNA extraction efficiency and reaction performance of the sample, an internal control (IC) was designed, which is a synthetic sequence with extremely low sequence homology and will not cross react with other genomes. A pair of primers was also designed for IC. The primers and the probes (Table 1) were synthesized by TBA (Tohoku Bio-Array, TBA, Sendai, Japan).

### 2.5. Establishment of Multiplex PCR-Dipstick DNA Chromatography

#### 2.5.1. Multiplex PCR Reaction

The reaction system includes 2.5 *μ*L 10 × PCR buffer, 5 *μ*L DNA template, 0.5 *μ*L forward and reverse primers of MP and CP (10 *μ*M), respectively, 0.5 *μ*L IC forward and IC reverse primers (2 *μ*M), respectively, 1 *μ*L IC DNA (1 nM), 1.2 *μ*L RT enzyme mixture, 1 *μ*L dUTP + dNTP mixture, 0.02 *μ*L uracil N-glycosylase (UNG), 0.3 *μ*L Taq HS polymerase, and 10.98 *μ*L deionized water, in a total volume of 25 *μ*L. The PCR amplification reaction procedure was performed at 95°C for 3 minutes, and the amplification programme for a total of 40 cycles is as follows: 95°C for 5 s; 60°C for 20 s.

#### 2.5.2. Dipstick DNA Chromatography

**Figure 1:**
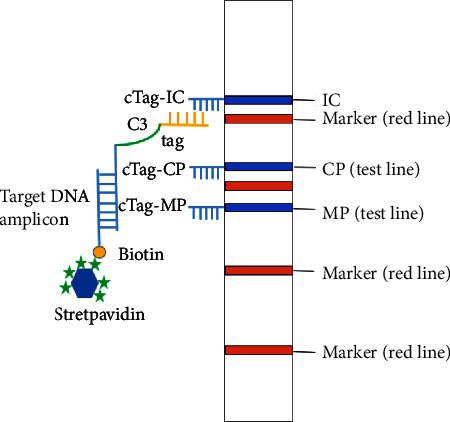
The schematic diagram of multiplex PCR-dipstick DNA chromatography.

The dipstick strip, manufactured by Tohoku Bio-Array, Co., contains three primary components: a polyvinyl chloride (PVC) membrane provides stability and adhesiveness, an absorbent area made of absorbent filter paper, and a sample application area made of a nitrocellulose (NC) membrane. Complementary oligonucleotide probes (cTag) were immobilized on an NC membrane to form M. pneumoniae and C. pneumoniae test lines and an internal control line. In addition, the sample application area was printed with four red position lines. The red line is used as a marker line to locate different bacterial test lines. Biotin at the 5′end of the reverse primer combined with the blue latex microspheres coated with streptavidin to generate signals. Target DNA amplicons with Tag and a biotinylated terminus were coupled with latex beads through a streptavidin-biotin interaction, and then, Tag hybridized with cTag on the strip. The accumulation of captured latex beads on the test line and IC line produced blue bands, enabling visual detection with the naked eye. After PCR amplification, 10 *μ*L PCR products, 9 *μ*L eluent (containing detergent, blocking agent, PBS, and salt solution), and 1 *μ*L of streptavidin-coated blue latex were added to the test tube to form a mixture, and then, the test strip was inserted into the mixture. After 10 minutes, the corresponding test lines and IC lines turn blue, indicating that the target bacteria are positive. If only the IC line shows color, it is negative. If the IC line does not show color, it indicates that the test strip is invalid. The schematic diagram of multiplex PCR-dipstick DNA chromatography is shown in Figure 1.

### 2.6. Specificity of Multiplex PCR-Dipstick Chromatography

The PCR-dipstick chromatography assay was utilized to detect a concentration of 105CFU/mL of various bacterial pathogens, including *M. pneumoniae*, *C. pneumoniae*, *Escherichia coli*, *Klebsiella pneumoniae*, *Pseudomonas aeruginosa*, *Haemophilus influenzae*, *Streptococcus pneumoniae*, *Staphylococcus aureus*, *Acinetobacter baumannii*, *Acinetobacter johnsonii*, *Enterobacteriaceae cloacae*, *Klebsiella aerogenes*, *Haemophilus parainfluenzae*, *Pseudomonas fluorescens*, *Aeromonas hydrophila*, *Staphylococcus epidermidis*, and *Chlamydia psittaci* to assess the specificity of this assay.

### 2.7. Sensitivity of Multiplex PCR-Dipstick Chromatography

In order to assess the limit of detection (LOD) of the PCR-dipstick chromatography assay, serial 10-fold dilutions of M. pneumoniae and C. pneumoniae strain DNA (10–10^7^ CFU/mL) were analyzed by the PCR-dipstick DNA chromatograph assay to determine the sensitivity, and the lowest concentration with a visible blue band at the corresponding detection line was the LOD of the PCR-dipstick chromatography assay.

### 2.8. Clinical Sample Validation

300 nasopharyngeal swab samples were detected by the PCR-dipstick chromatography assay, and the PCR products were sent for sequencing. The PCR-dipstick chromatography detection results were compared with MP-IgM, CP-IgM, and DNA sequencing, respectively. The diagnostic sensitivity, specificity, positive predictive value, and negative predictive value of the PCR-dipstick chromatography assay were evaluated with DNA sequencing as the gold standard. We used the SPSS pairing *χ*^2^ test (McNemar test) to compare the diagnostic efficiency of the PCR-dipstick chromatography assay with MP-IgM, CP-IgM, and DNA sequencing, respectively. *P* < 0.05 indicates a statistically significant difference. Kappa ≥ 0.7 indicates strong fit; 0.7 > kappa ≥ 0.4 indicates average fit; kappa < 0.4 indicates weak fit.

## 3. Results

### 3.1. Specificity of Multiplex PCR-Dipstick Chromatography

**Figure 2:**
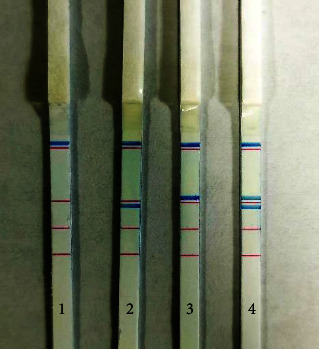
Specificity of multiplex PCR-dipstick chromatography. 1: negative control; 2: *M. pneumoniae*; 3: *C. pneumoniae*; 4: joint detection of *M. pneumoniae* and *C. pneumoniae*.

We detected *M. pneumoniae*, *C. pneumoniae*, and 15 pathogens (*Escherichia coli*, *Klebsiella pneumoniae*, *Pseudomonas aeruginosa*, *Haemophilus influenzae*, *Streptococcus pneumoniae*, *Staphylococcus aureus*, *Acinetobacter baumannii*, *Acinetobacter johnsonii*, *Enterobacteriaceae cloacae*, *Klebsiella aerogenes*, *Haemophilus parainfluenzae*, *Pseudomonas fluorescens*, *Aeromonas hydrophila*, *Staphylococcus epidermidis*, and *Chlamydia psittaci*) by the PCR-dipstick chromatography assay to evaluate the specificity of PCR-dipstick chromatography. There are visible blue bands in the corresponding test line for detection of *M. pneumoniae* and *C. pneumoniae*, but none of nonspecific amplification for 15 pathogens, consistent with negative results, implying no cross reaction with the 15 pathogens. Multiplex PCR-dipstick chromatography was applied to detect M. pneumoniae and C. pneumoniae strains' equivalent mixture (10^5^ CFU/mL), and M. pneumoniae and C. pneumoniae joint detection was observed (Figure 2). The results illustrated multiplex PCR-dipstick chromatography has good specificity and no cross reactivity with other pathogens.

### 3.2. Sensitivity of Multiplex PCR-Dipstick Chromatography

**Figure 3:**
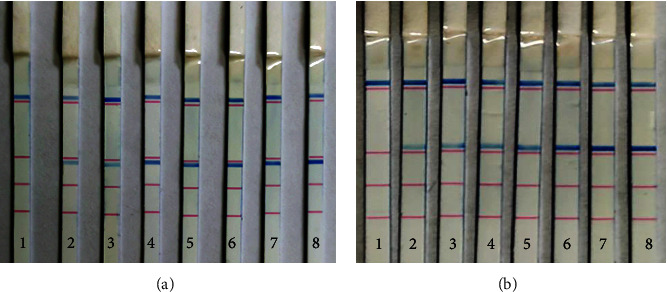
Sensitivity of the multiplex PCR-dipstick chromatography assay for detection of *M. pneumoniae* and *C. pneumoniae*. (a) *M. pneumoniae*; (b) *C. pneumoniae*. 1: negative control; 2–8: detection of each bacterial standard strain DNA with a concentration ranging from 10 CFU/mL to 10^7^ CFU/mL, respectively.

In order to assess the limit of detection (LOD) of multiplex PCR-dipstick chromatography, serial 10-fold dilutions of *M. pneumoniae* and *C. pneumoniae* (10–10^7^ CFU/mL) were analyzed by multiplex PCR-dipstick DNA chromatography to determine the sensitivity. M. pneumoniae and C. pneumoniae sensitivity of the PCR-dipstick chromatography assay was 10 CFU/mL, respectively (Figure 3).

### 3.3. Data of Clinical Specimens

Nasopharyngeal swab samples of 300 children with an acute respiratory tract infection were detected by the multiplex PCR-dipstick chromatography assay and DNA sequencing. Meanwhile, blood samples from each child were identified by the MP-IgM and CP-IgM antibody assay. As shown in Tables 2 and 3, among the samples from children with an acute respiratory tract infection, 53 (17.7%) of them were positive for MP, 16 (5.3%) of them were positive for CP, and 4 (1.3%) of them were simultaneously positive for MP and CP by the multiplex PCR-dipstick chromatography assay. DNA sequencing showed that 55 cases (18.3%) were MP positive, 17 cases (5.7%) were CP positive, and 4 cases (1.3%) were simultaneously positive for MP and CP. There was no statistical significance for MP and CP detection between the multiplex PCR-dipstick chromatography assay and DNA sequencing (MP: *P*=0.5; CP: *P*=1.0), and a high statistical consistency was observed between the two assays (MP: kappa value = 0.979; CP: kappa value = 0.974).

Blood samples of all children were tested by the MP-IgM and CP-IgM assay. Among 300 samples, 40 cases (13.3%) were positive for MP-IgM, 10 cases (3.3%) were positive for CP-IgM, and 2 cases (0.67%) were simultaneously positive for MP-IgM and CP-IgM. The multiplex PCR-dipstick chromatography assay for detecting MP and CP showed statistical significance compared to serum MP-IgM and CP-IgM, with *P*=0.001 (MP) and *P*=0.021 (CP), respectively. The two assays for detecting MP have a high degree of agreement, with a kappa value of 0.747. The agreement for detecting CP is average, with a kappa value of 0.671.

## 4. Discussion

Atypical pneumonia caused by *M. pneumoniae* and *C. pneumoniae* has similar respiratory manifestations to bacterial and viral infections, which is prone to misdiagnosis and missed diagnosis. Therefore, rapid, sensitive, and specific detection methods are necessary for clinical diagnosis. In order to improve laboratory diagnosis and reduce the cost and time of detecting *M. pneumoniae* and *C. pneumoniae*, we designed and validated a multiplex PCR-dipstick chromatography assay that can simultaneously detect *M. pneumoniae* and *C. pneumoniae*. The results indicated that we successfully developed a multiplex PCR-dipstick chromatography assay in this study to achieve rapid detection of *M. pneumoniae* and *C. pneumoniae* within 2 hours. Real-time fluorescence quantitative PCR detection is often used to identify *M. pneumoniae* due to its high sensitivity and specificity [22, 23]. The limit of detection (LOD) of multiplex PCR-dipstick chromatography for *M. pneumoniae* and *C. pneumoniae* was 10 CFU/mL, which is not lower than that of real-time fluorescence quantitative PCR. In addition, this assay has strong specificity and does not require complex and expensive equipment. It only requires a regular PCR amplification instrument, which has broad prospects for promotion and application. Using DNA sequencing as the diagnostic gold standard, 300 clinical samples were analyzed by multiplex PCR-dipstick chromatography. The sensitivity, specificity, positive predictive value, and negative predictive value of the multiplex PCR-dipstick chromatography assay for the diagnosis of *M. pneumoniae* were 96.61%, 100%, 100%, and 99.18%, respectively, and the sensitivity, specificity, positive predictive value, and negative predictive value for the diagnosis of *C. pneumoniae* were 95.24%, 100%, 100%, and 99.64%, respectively. The positive rate of the multiplex PCR-dipstick chromatography assay was significantly higher than that of the serum IgM antibody assay, with *M. pneumoniae* (17.7% vs. 13.3%), *C. pneumoniae* (5.7% vs. 3.3%), and mixed infection of *M. pneumoniae* and *C. pneumoniae* (1.3% vs. 0.67%). For 57 positive cases of *M. pneumoniae* detected by the multiplex PCR-dipstick chromatography assay, only 39 cases were MP-IgM positive and 18 cases were MP-IgM negative. Among the 20 positive cases of CP detected by the multiplex PCR-dipstick chromatography assay, 11 cases were CP-IgM positive and 9 cases were CP-IgM negative. Reviewing the case data of 18 patients with negative MP-IgM and 9 patients with negative CP-IgM, 12 cases of the 18 patients with negative MP-IgM had a disease course of less than one week and 6 cases had a disease course of 7–10 days. Among the 9 patients with negative CP-IgM, 6 cases had a course of less than one week, 2 had a course of 8-9 days, and 1 case had a course of 12 days. Due to the increase of IgM in about one week of onset and reaches its peak in 3–6 weeks [11], the inconsistency between the multiplex PCR-dipstick chromatography assay and serum IgM antibody levels may stem from the presence of an antibody production window during serum antibody testing. This window refers to a specific timeframe in which the body either does not produce antibodies or produces them in such low concentrations that they may not be detected. In this study, three cases of MP IgM positive and one case of CP IgM positive were identified, however, all four samples showed negative results by multiplex PCR-dipstick chromatography assay. To further investigate this discrepancy, DNA sequencing was performed on the four samples. Only one sample that had tested positive for MP IgM yielded a positive result through DNA sequencing. This finding suggested that the inconsistency observed in this case might be attributed to the low concentration of the bacterium in the nasopharyngeal swab samples, which was below the detection limit of the multiplex PCR-dipstick chromatography assay. On the other hand, the remaining two cases of MP IgM positive and one case of CP IgM positive, which had shown negative results by multiplex PCR-dipstick chromatography, were further confirmed as negative through DNA sequencing. Their inconsistency results between multiplex PCR-dipstick chromatography assay and serum IgM antibody was believed as improper sampling technique leading to false-negative results of PCR-dipstick chromatography assay. The multiplex PCR-dipstick chromatography assay and DNA sequencing showed a high statistical consistency between MP and CP (MP: kappa value = 0.979; CP: kappa value = 0.974). The results indicated that the multiplex PCR-dipstick chromatography assay is an effective and valuable diagnostic tool for clinical detection of *M. pneumoniae* and *C. pneumoniae* in the early stages of infection.

Remaining DNA can last for 8 months or even longer after M. pneumoniae and C. pneumoniae infection. Thus, the obvious limitation of the multiplex PCR-dipstick chromatography assay is that it can only be used for early infection diagnosis but not suitable for determining the clearance rate of pathogens after medication. A positive result cannot distinguish between carriers and recent or past infection status. Second, macrolide-resistant strains of *M. pneumoniae* and *C. pneumoniae* have emerged in the past few years, with a high incidence rate in China and Japan, which may lead to treatment failure. Therefore, the detection of resistance to macrolide drugs is also crucial, but this assay cannot determine drug resistance.

With the development of the signal processing, probe, and multiplex PCR technology of the strip biosensor, resistance genes to macrolide antibiotics should be incorporated in a PCR-dipstick chromatography assay.

## 5. Conclusion

This study successfully established a multiplex PCR-dipstick chromatography assay for the joint detection of *M. pneumoniae* and *C. pneumoniae*. It is fast, sensitive, and specific and is expected to become a new technical means for clinical diagnosis and rapid screening, which is suitable for small laboratories and point-of-care diagnosis.

## Figures and Tables

**Table 1:** The primers for the PCR-dipstick DNA chromatography assay.

Primers	Gene	Tag	Sequence (5′-3′)	Amplicon size (bp)
MP-F	RI3	tagctaagtggtccataact	Tag-C3-AAGGAAGCTGACTCCGACAA	107
MP-R			Biotin-CCAAACACCAAAAGCGCTTG	

CP-F	AR39	ctgcgggtatagaagccct	Tag-C3-GCACTTTCATGGGAGCCTTT	92
CP-R			Biotin-GCGCATTCCAAAAGCTTCAC	

Internal control: F	NM-005157	gctctagccaccaatgaatctaa	Tag IC-C3-CAGAGCACAGAGACACCACT	153
Internal control: R			Biotin-GGCGCTCATCTTCATTCAGG	

F: forward primer; R: reverse primer.

**Table 2:** Comparison of clinical specimens of MP data between multiplex PCR-dipstick chromatography assay, DNA sequencing, and MP-IgM.

PCR-dipstickchromatography	DNA sequencing		Total	*P* value	MP-IgM		Total	*P* value
	Positive	Negative		Kappa value	Positive	Negative		Kappa value
Positive	57	0	57	*P*=0.5	39	18	57	*P*=0.001
Negative	2	241	243	Kappa = 0.979	3	240	243	Kappa = 0.747
Total	59	241	300		42	258	300	

**Table 3:** Comparison of clinical specimens of CP data between multiplex PCR-dipstick chromatography assay, DNA sequencing, and CP-IgM.

PCR-dipstickchromatography	DNA sequencing		Total	*P* value	CP-IgM		Total	*P* value
	Positive	Negative		Kappa value	Positive	Negative		Kappa value
Positive	20	0	20	*P*=1.0	11	9	20	*P*=0.021
Negative	1	279	280	Kappa = 0.974	1	279	280	Kappa = 0.671
Total	21	279	300		12	288	300	

## Data Availability

The data used to support the findings of this study are available from the corresponding author upon request.

## Supplementary Materials

Supplementary Materials We have uploaded a Word file named “Clinical Sample Test Results” to Supplementary Materials, which contain MP-IgM and CP-IgM S/CO values and corresponding multiplex PCR-dipstick DNA chromatography results. Five images were uploaded to Supplementary Materials, which contain the blue intensity of 53cases positive for MP, 16 cases positive for CP, and 4 cases simultaneously positive for MP and CP by the multiplex PCR-dipstick chromatography assay.
