# Supplementary material for: Establishment and Performance Evaluation of Multiplex PCR-Dipstick DNA Chromatography for Mycoplasma pneumoniae and Chlamydia pneumoniae Rapid Detection
Source: Can J Infect Dis Med Microbiol. 2023 Sep 28;2023:6654504. doi: 10.1155/2023/6654504 (PMC10555492; doi:10.1155/2023/6654504)
Supplement: Supplementary Materials — We have uploaded a Word file named “Clinical Sample Test Results” to Supplementary Materials, which contain MP-IgM and CP-IgM S/CO values and corresponding multiplex PCR-dipstick DNA chromatography results. Five images were uploaded to Supplementary Materials, which contain the blue intensity of 53cases positive for MP, 16 cases positive for CP, and 4 cases simultaneously positive for MP and CP by the multiplex PCR-dipstick chromatography assay. [file 6654504.f1.zip › Clinical Sample Test Results.docx]

| Number | Patient ID | sex | age | CP-Ig M S/CO | MP-Ig M S/CO | multiplex PCR-Dipstick DNA Chromatography-CP | multiplex PCR-Dipstick DNA Chromatography-MP |
| --- | --- | --- | --- | --- | --- | --- | --- |
| 1 | 02988971 | W | 7year | 0.0 | 4.3 | - | + |
| 2 | 90520043 | W | 2year | 0.2 | 1.5 | - | + |
| 3 | 90271295 | W | 4year | 0.1 | 1.8 | - | + |
| 4 | 03708479 | M | 4year | 0.1 | 1.0 | - | + |
| 5 | 03245663 | M | 6 year | 0.0 | 1.0 | - | + |
| 6 | 90628974 | M | 2year | 0.1 | 5.7 | - | + |
| 7 | 03905238 | W | 2year | 0.1 | 1.3 | - | + |
| 8 | 02470541 | W | 8year | 0.1 | 1.0 | - | + |
| 9 | 04121280 | W | 3year | 0.1 | 1.2 | - | + |
| 10 | 91170117 | M | 3year | 0.1 | 1.0 | - | + |
| 11 | 90410532 | W | 3year | 0.0 | 1.9 | - | + |
| 12 | 03483081 | W | 8year | 0.1 | 38.4 | - | + |
| 13 | 90261187 | W | 3year | 0.5 | 1.0 | - | - |
| 14 | 90712229 | M | 6year | 0.1 | 1.1 | - | + |
| 15 | 03353359 | W | 5year | 0.0 | 7.6 | - | + |
| 16 | 90292761 | M | 2year | 0.0 | 1.2 | - | + |
| 17 | 90238768 | W | 3year | 0.0 | 1.5 | - | + |
| 18 | 90701313 | M | 5year | 0.0 | 6.5 | - | + |
| 19 | 03975087 | W | 3year | 0.1 | 1.2 | - | + |
| 20 | 90128363 | M | 8year | 0.0 | 1.3 | - | + |
| 21 | 04037627 | M | 2year | 0.1 | 1.0 | - | - |
| 22 | 03809476 | W | 3year | 0.0 | 1.1 | - | + |
| 23 | 04128430 | M | 1year | 0.1 | 1.2 | - | + |
| 24 | 90949643 | W | 9year | 0.1 | 2.2 | - | + |
| 25 | 03815934 | W | 2year | 0.0 | 1.5 | - | + |
| 26 | 03436028 | W | 4year | 0.0 | 1.0 | - | - |
| 27 | 03604068 | M | 4year | 0.1 | 5.3 | - | + |
| 28 | 90117075 | W | 4 year | 0.3 | 1.3 | - | + |
| 29 | 91213864 | M | 3year | 0.1 | 2.2 | - | + |
| 30 | 03753653 | M | 1year | 0.1 | 1.8 | - | + |
| 31 | 03676498 | W | 5year | 0.5 | 1.2 | - | + |
| 32 | 90857056 | W | 2year | 0.0 | 2.1 | - | + |
| 33 | 90614048 | W | 3year | 0.1 | 1.6 | - | + |
| 34 | 90799473 | M | 4year | 0.1 | 2.3 | - | + |
| 35 | 91197525 | M | 2year | 0.1 | 1.1 | - | + |
| 36 | 03808075 | M | 3 year | 0.0 | 6.9 | - | + |
| 37 | 03501647 | M | 5year | 0.0 | 22.6 | - | + |
| 38 | 02314158 | M | 3year | 0.1 | 1.3 | - | + |
| 39 | 90451272 | W | 2year | 0.0 | 1.1 | - | + |
| 40 | 03131701 | M | 6year | 0.0 | 3.7 | - | + |
| 51 | 02869330 | W | 7year | 9.7 | 3.8 | + | + |
| 52 | 03271836 | W | 6year | 5.2 | 7.5 | + | + |
| 41 | 90069066 | M | 8 year | 5.1 | 0.2 | + | - |
| 42 | 02221429 | M | 10year | 9.1 | 0.4 | + | - |
| 43 | 03542590 | M | 7year | 1.0 | 0.3 | - | - |
| 44 | 91127997 | W | 13 year | 1.7 | 0.9 | + | + |
| 45 | 91136516 | M | 10 year | 3.6 | 0.9 | + | + |
| 46 | 90228642 | W | 3 year | 2.3 | 0.4 | + | - |
| 47 | 91117099 | W | 11year | 2.8 | 0.9 | + | - |
| 48 | 03847984 | W | 3year | 1.4 | 0.2 | + | - |
| 49 | 02221671 | M | 10year | 8.2 | 0.8 | + | - |
| 50 | 91117099 | W | 11year | 2.4 | 0.9 | + | - |
| 53 | 02797469 | W | 8year | 0.4 | 0.9 | - | - |
| 54 | 03935227 | M | 2year | 0.1 | 0.2 | - | - |
| 55 | 90769829 | M | 1year | 0.0 | 0.2 | - | - |
| 56 | 90385767 | M | 3year | 0.0 | 0.4 | - | - |
| 57 | 90765441 | W | 1year | 0.0 | 0.2 | - | - |
| 58 | 03590161 | M | 5year | 0.1 | 0.2 | - | - |
| 59 | 91068226 | W | 1year | 0.0 | 0.6 | - | - |
| 60 | 90776623 | W | 1year | 0.1 | 0.2 | - | - |
| 61 | 03759350 | M | 4year | 0.1 | 0.2 | - | - |
| 62 | 03375152 | M | 4year | 0.4 | 0.2 | - | - |
| 63 | 91203084 | W | 1year | 0.0 | 0.4 | - | - |
| 64 | 90377751 | M | 3year | 0.0 | 0.3 | - | - |
| 65 | 90732075 | W | 3year | 0.2 | 0.2 | - | - |
| 66 | 03352399 | W | 6year | 0.2 | 0.8 | - | - |
| 67 | 03247762 | W | 5year | 0.5 | 0.5 | + | - |
| 68 | 90199657 | W | 3year | 0.1 | 0.5 | - | - |
| 69 | 04199451 | W | 5月 | 0.0 | 0.2 | - | - |
| 70 | 90622926 | W | 3year | 0.1 | 0.3 | - | - |
| 71 | 03441003 | M | 5year | 0.0 | 0.1 | - | - |
| 72 | 90281516 | W | 3year | 0.0 | 0.5 | - | - |
| 73 | 03747456 | M | 7year | 0.4 | 0.5 | + | - |
| 74 | 03595924 | M | 4year | 0.1 | 0.2 | - | - |
| 75 | 90536224 | M | 2year | 0.1 | 0.7 | - | - |
| 76 | 90401223 | W | 2year | 0.0 | 0.4 | - | - |
| 77 | 03061530 | M | 6year | 0.3 | 0.9 | - | - |
| 78 | 90262044 | M | 3year | 0.0 | 0.1 | - | - |
| 79 | 03753684 | M | 6月 | 0.0 | 0.1 | - | - |
| 80 | 90795782 | M | 3year | 0.1 | 0.2 | - | - |
| 81 | 90218710 | M | 4year | 0.1 | 0.6 | - | - |
| 82 | 90249345 | W | 3year | 0.2 | 0.5 | - | - |
| 83 | 04122544 | M | 2year | 0.0 | 0.1 | - | - |
| 84 | 90343026 | M | 2year | 0.1 | 0.2 | - | - |
| 85 | 50020180 | M | 5year | 0.1 | 0.6 | - | - |
| 86 | 04136669 | M | 1year | 0.0 | 0.1 | - | - |
| 87 | 03463556 | M | 5year | 0.7 | 0.1 | + | - |
| 88 | 03839163 | M | 3year | 0.0 | 0.1 | - | - |
| 89 | 91051758 | W | 1 year | 0.1 | 0.4 | - | - |
| 90 | 03847895 | M | 3year | 0.0 | 0.1 | - | - |
| 91 | 90667619 | W | 4year | 0.1 | 0.5 | - | + |
| 92 | 90952970 | M | 4year | 0.0 | 0.1 | - | - |
| 93 | 90703576 | M | 1year | 0.1 | 0.5 | - | + |
| 94 | 90893561 | W | 6year | 0.1 | 0.3 | - | + |
| 95 | 90407708 | W | 3year | 0.0 | 0.2 | - | + |
| 96 | 03679507 | M | 4year | 0.1 | 0.7 | - | + |
| 97 | 90613472 | M | 3year | 0.0 | 0.4 | - | - |
| 98 | 91204540 | M | 3year | 0.1 | 0.2 | - | - |
| 99 | 91013324 | W | 4year | 0.1 | 0.3 | - | - |
| 100 | 90458924 | W | 1year | 0.1 | 0.7 | - | + |
| 101 | 90046650 | W | 6 year | 0.1 | 0.2 | - | + |
| 102 | 03709066 | W | 4year | 0.0 | 0.6 | - | - |
| 103 | 03554578 | M | 6year | 0.0 | 0.2 | - | - |
| 104 | 91213710 | W | 3year | 0.0 | 0.7 | - | + |
| 105 | 91192353 | M | 3year | 0.0 | 0.2 | - | - |
| 106 | 91146049 | M | 6月 | 0.0 | 0.2 | - | - |
| 107 | 90282227 | W | 3year | 0.0 | 0.5 | - | - |
| 108 | 90445699 | W | 2year | 0.1 | 0.1 | - | - |
| 109 | 03281894 | M | 5year | 0.1 | 0.8 | - | + |
| 110 | 90221342 | M | 3year | 0.0 | 0.9 | - | + |
| 111 | 90975054 | M | 10月 | 0.0 | 0.6 | - | + |
| 112 | 91156357 | M | 4year | 0.0 | 0.4 | - | - |
| 113 | 91213890 | W | 6year | 0.1 | 0.3 | - | - |
| 114 | 90294765 | W | 4year | 0.1 | 0.5 | - | - |
| 115 | 90527538 | M | 3year | 0.1 | 0.7 | - | - |
| 116 | 90919658 | M | 1year | 0.0 | 0.3 | - | - |
| 117 | 03702028 | M | 6year | 0.2 | 0.5 | - | - |
| 118 | 03847944 | M | 3year | 0.1 | 0.6 | - | - |
| 119 | 90338633 | M | 2year | 0.0 | 0.8 | - | - |
| 120 | 03967262 | W | 1year | 0.0 | 0.3 | - | - |
| 121 | 90301826 | M | 3year | 0.0 | 0.4 | - | - |
| 122 | 90101101 | M | 4year | 0.0 | 0.4 | - | - |
| 123 | 90801817 | M | 1year | 0.0 | 0.4 | - | - |
| 124 | 90291100 | W | 2year | 0.0 | 0.6 | - | - |
| 125 | 03828563 | M | 3year | 0.0 | 0.4 | - | - |
| 126 | 03976122 | M | 2year | 0.0 | 0.3 | - | - |
| 127 | 91159469 | M | 2year | 0.0 | 0.2 | - | - |
| 128 | 90127187 | W | 6year | 0.1 | 0.6 | + | - |
| 129 | 90460258 | M | 3year | 0.0 | 0.5 | - | - |
| 130 | 50003509 | M | 4year | 0.0 | 0.5 | - | - |
| 131 | 90826772 | W | 4year | 0.1 | 0.4 | - | - |
| 132 | 03754494 | M | 6月 | 0.0 | 0.2 | - | - |
| 133 | 03939224 | M | 2year | 0.1 | 0.7 | - | - |
| 134 | 90411878 | W | 2year | 0.0 | 0.4 | - | - |
| 135 | 04009036 | M | 2year | 0.1 | 0.5 | - | - |
| 136 | 91215765 | M | 2 year | 0.1 | 0.8 | - | - |
| 137 | 03041052 | M | 3year | 0.1 | 0.7 | - | - |
| 138 | 90290698 | M | 3year | 0.0 | 0.9 | - | + |
| 139 | 03889677 | M | 3year | 0.0 | 0.3 | - | - |
| 140 | 04180980 | M | 9月 | 0.0 | 0.7 | - | - |
| 141 | 90263233 | M | 3year | 0.1 | 0.7 | - | - |
| 142 | 03542978 | M | 6year | 0.0 | 0.6 | - | - |
| 143 | 03455751 | W | 5year | 0.1 | 0.7 | - | - |
| 144 | 90845599 | M | 3year | 0.0 | 0.7 | - | - |
| 145 | 90309984 | M | 2year | 0.1 | 0.5 | - | - |
| 146 | 90474126 | M | 2year | 0.1 | 0.3 | - | - |
| 147 | 03855196 | M | 4year | 0.0 | 0.2 | - | - |
| 148 | 90416459 | M | 3year | 0.0 | 0.3 | - | - |
| 149 | 90459774 | M | 2year | 0.1 | 0.8 | - | + |
| 150 | 02854935 | W | 8year | 0.1 | 0.4 | - | - |
| 151 | 03581448 | W | 2year | 0.0 | 0.4 | - | - |
| 152 | 03582283 | M | 3year | 0.0 | 0.2 | - | - |
| 153 | 90076077 | W | 5year | 0.1 | 0.7 | - | + |
| 154 | 03742259 | W | 2year | 0.0 | 0.4 | - | - |
| 155 | 91024836 | W | 3year | 0.0 | 0.5 | - | + |
| 156 | 91105814 | M | 4year | 0.0 | 0.7 | - | - |
| 157 | 03837624 | M | 3year | 0.0 | 0.2 | - | - |
| 158 | 50016151 | M | 6year | 0.1 | 0.4 | - | - |
| 159 | 04106611 | W | 3year | 0.0 | 0.4 | - | + |
| 160 | 03903018 | M | 3year | 0.3 | 0.7 | - | - |
| 161 | 02871344 | W | 7year | 0.1 | 0.2 | - | - |
| 162 | 90105392 | W | 4year | 0.0 | 0.5 | - | - |
| 163 | 90729868 | M | 4year | 0.4 | 0.6 | - | - |
| 164 | 90715917 | W | 1year | 0.1 | 0.6 | - | - |
| 165 | 03582816 | W | 4year | 0.2 | 0.9 | - | - |
| 166 | 90425920 | W | 2year | 0.2 | 0.6 | - | - |
| 167 | 90365559 | M | 3year | 0.0 | 0.5 | - | - |
| 168 | 03905674 | M | 3year | 0.0 | 0.6 | - | - |
| 169 | 90109906 | W | 5year | 0.1 | 0.4 | - | - |
| 170 | 03714563 | M | 4year | 0.1 | 0.6 | - | - |
| 171 | 90708776 | W | 4year | 0.0 | 0.5 | - | - |
| 172 | 90811528 | M | 5year | 0.2 | 0.4 | - | - |
| 173 | 90319123 | M | 3year | 0.0 | 0.3 | - | - |
| 174 | 50038863 | M | 4year | 0.0 | 0.9 | - | - |
| 175 | 90990758 | W | 3year | 0.1 | 0.4 | - | - |
| 176 | 03847971 | W | 3year | 0.0 | 0.3 | - | - |
| 177 | 03662446 | M | 4year | 0.0 | 0.4 | - | - |
| 178 | 04155986 | M | 3year | 0.0 | 0.2 | - | - |
| 179 | 90761729 | W | 4year | 0.0 | 0.5 | - | - |
| 180 | 03590569 | W | 4year | 0.2 | 0.6 | - | - |
| 181 | 91074813 | W | 3year | 0.0 | 0.4 | - | - |
| 182 | 90427635 | M | 3 year | 0.0 | 0.4 | - | - |
| 183 | 90547629 | M | 1year | 0.0 | 0.4 | - | - |
| 184 | 90539518 | W | 1year | 0.0 | 0.9 | - | - |
| 185 | 03903634 | M | 3year | 0.0 | 0.5 | - | - |
| 186 | 91046444 | M | 3year | 0.0 | 0.3 | - | - |
| 187 | 04046023 | M | 9月 | 0.0 | 0.7 | - | - |
| 188 | 90254479 | M | 3year | 0.0 | 0.5 | - | - |
| 189 | 90275992 | M | 3year | 0.0 | 0.3 | - | - |
| 190 | 90272660 | M | 3year | 0.0 | 0.3 | - | - |
| 191 | 50043474 | M | 2year | 0.1 | 0.4 | - | - |
| 192 | 90630817 | M | 5year | 0.1 | 0.4 | - | - |
| 193 | 91161938 | M | 3year | 0.1 | 0.8 | - | - |
| 194 | 50029825 | M | 4year | 0.0 | 0.5 | - | - |
| 195 | 90270971 | W | 4year | 0.0 | 0.3 | - | - |
| 196 | 03724797 | M | 4year | 0.0 | 0.7 | - | - |
| 197 | 02442541 | M | 9year | 0.0 | 0.2 | - | - |
| 198 | 03747025 | M | 4 year | 0.0 | 0.5 | - | - |
| 199 | 04061688 | W | 11月 | 0.0 | 0.4 | - | - |
| 200 | 02590312 | M | 9year | 0.0 | 0.2 | - | - |
| 201 | 02788779 | M | 8year | 0.1 | 0.9 | + | - |
| 202 | 50011696 | M | 6year | 0.0 | 0.3 | - | - |
| 203 | 91230013 | W | 4year | 0.0 | 0.7 | - | - |
| 204 | 91195913 | M | 3year | 0.1 | 0.4 | - | - |
| 205 | 91176132 | W | 3year | 0.0 | 0.4 | - | - |
| 206 | 91214222 | M | 2year | 0.1 | 0.8 | - | - |
| 207 | 91201479 | W | 6year | 0.1 | 0.4 | - | - |
| 208 | 03746552 | M | 3year | 0.0 | 0.3 | - | - |
| 209 | 91107685 | W | 4year | 0.2 | 0.8 | + | - |
| 210 | 80017964 | M | 4year | 0.0 | 0.3 | - | - |
| 211 | 04027467 | M | 2year | 0.0 | 0.7 | - | - |
| 212 | 03354168 | W | 6year | 0.0 | 0.4 | - | - |
| 213 | 33009612 | M | 8year | 0.0 | 0.6 | - | - |
| 214 | 03704289 | M | 5year | 0.0 | 0.6 | - | - |
| 215 | 04156756 | M | 2year | 0.0 | 0.3 | - | - |
| 216 | 90759154 | M | 3year | 0.0 | 0.3 | - | - |
| 217 | 90597394 | M | 1year | 0.0 | 0.8 | - | - |
| 218 | 03441221 | M | 5year | 0.0 | 0.2 | - | - |
| 219 | 90646894 | M | 8year | 0.0 | 0.4 | - | - |
| 220 | 90193669 | M | 3year | 0.0 | 0.9 | - | - |
| 221 | 91227658 | W | 3月 | 0.0 | 0.2 | - | - |
| 222 | 90956268 | W | 4 year | 0.0 | 0.3 | - | - |
| 223 | 90454245 | M | 5year | 0.0 | 0.4 | - | - |
| 224 | 50002053 | M | 6year | 0.0 | 0.5 | - | - |
| 225 | 90072373 | M | 4year | 0.0 | 0.9 | - | - |
| 226 | 90162941 | M | 3year | 0.0 | 0.4 | - | - |
| 227 | 90126413 | W | 4year | 0.2 | 0.8 | - | - |
| 228 | 90273951 | W | 3year | 0.1 | 0.4 | - | - |
| 229 | 90291847 | M | 2year | 0.2 | 0.4 | - | - |
| 230 | 04090789 | M | 1year | 0.0 | 0.6 | - | - |
| 231 | 90212852 | W | 3year | 0.2 | 0.9 | - | - |
| 232 | 03352860 | M | 5year | 0.1 | 0.4 | - | - |
| 233 | 03620330 | W | 4year | 0.1 | 0.3 | - | - |
| 234 | 90529473 | W | 3year | 0.1 | 0.8 | - | - |
| 235 | 90282341 | W | 3year | 0.1 | 0.5 | - | - |
| 236 | 90738772 | M | 4year | 0.1 | 0.8 | - | - |
| 237 | 04038177 | W | 3year | 0.0 | 0.7 | - | - |
| 238 | 90490901 | W | 3year | 0.1 | 0.6 | - | - |
| 239 | 90984032 | W | 2year | 0.1 | 0.9 | - | - |
| 240 | 90282147 | W | 3year | 0.2 | 0.9 | - | - |
| 241 | 03966297 | M | 11月 | 0.0 | 0.6 | - | - |
| 242 | 04061300 | M | 1year | 0.0 | 0.2 | - | - |
| 243 | 02511820 | W | 9year | 0.1 | 0.3 | - | - |
| 244 | 91229488 | W | 5year | 0.1 | 0.8 | - | - |
| 245 | 03410559 | M | 5year | 0.0 | 0.4 | - | - |
| 246 | 04034509 | M | 1year | 0.0 | 0.3 | - | - |
| 247 | 91224996 | M | 3year | 0.0 | 0.4 | - | - |
| 248 | 90187135 | M | 4year | 0.0 | 0.7 | - | - |
| 249 | 91194557 | M | 2year | 0.0 | 0.6 | - | - |
| 250 | 90479285 | M | 2year | 0.0 | 0.4 | - | - |
| 251 | 04195616 | W | 4year | 0.1 | 0.6 | - | - |
| 252 | 03967966 | M | 1year | 0.0 | 0.5 | - | - |
| 253 | 90633427 | W | 5year | 0.0 | 0.6 | - | - |
| 254 | 04006584 | W | 5year | 0.0 | 0.3 | - | - |
| 255 | 90769084 | M | 1year | 0.0 | 0.9 | - | - |
| 256 | 03501194 | M | 5year | 0.0 | 0.3 | - | - |
| 257 | 91237383 | M | 3year | 0.0 | 0.5 | - | - |
| 258 | 90022834 | W | 5year | 0.2 | 0.8 | + | - |
| 259 | 03439985 | M | 5year | 0.1 | 0.9 | - | - |
| 260 | 90982890 | W | 1year | 0.0 | 0.6 | - | - |
| 261 | 91241496 | M | 2year | 0.1 | 0.6 | - | - |
| 262 | 90277335 | M | 3year | 0.2 | 0.5 | - | - |
| 263 | 02757264 | W | 4year | 0.0 | 0.5 | - | - |
| 264 | 90750066 | W | 1year | 0.0 | 0.5 | - | - |
| 265 | 04155766 | W | 4year | 0.1 | 0.2 | - | - |
| 266 | 91243305 | M | 4year | 0.0 | 0.3 | - | - |
| 267 | 90460563 | M | 3year | 0.0 | 0.4 | - | - |
| 268 | 02694189 | M | 8year | 0.0 | 0.5 | - | - |
| 269 | 90549431 | M | 1year | 0.0 | 0.5 | - | - |
| 270 | 90543054 | M | 2year | 0.1 | 0.5 | - | - |
| 271 | 04199406 | M | 4year | 0.0 | 0.2 | - | - |
| 272 | 03736128 | M | 7year | 0.0 | 0.4 | - | - |
| 273 | 90278702 | M | 3year | 0.1 | 0.2 | - | - |
| 274 | 90368180 | W | 2year | 0.0 | 0.6 | - | - |
| 275 | 91246426 | W | 11月 | 0.0 | 0.7 | - | - |
| 276 | 90443434 | W | 2year | 0.1 | 0.4 | - | - |
| 277 | 90453844 | M | 2year | 0.1 | 0.3 | - | - |
| 278 | 90646959 | M | 6year | 0.1 | 0.2 | - | - |
| 279 | 91167749 | M | 1year | 0.0 | 0.6 | - | - |
| 280 | 03270785 | W | 6year | 0.6 | 0.8 | + | - |
| 281 | 03925901 | M | 3year | 0.0 | 0.7 | - | - |
| 282 | 90918720 | W | 5year | 0.1 | 0.5 | - | - |
| 283 | 91203707 | W | 3月 | 0.0 | 0.2 | - | - |
| 284 | 06260626 | W | 3year | 0.0 | 0.4 | - | - |
| 285 | 03677931 | W | 7year | 0.1 | 0.5 | - | - |
| 286 | 90800081 | M | 3year | 0.1 | 0.6 | - | - |
| 287 | 90142825 | W | 5year | 0.0 | 0.2 | - | - |
| 288 | 90431711 | W | 2year | 0.0 | 0.2 | - | - |
| 289 | 03826191 | W | 3year | 0.0 | 0.4 | - | - |
| 290 | 03738527 | M | 3year | 0.0 | 0.9 | - | - |
| 291 | 03847935 | M | 3year | 0.0 | 0.3 | - | - |
| 292 | 03847936 | W | 3year | 0.0 | 0.3 | - | - |
| 293 | 03600020 | W | 4year | 0.0 | 0.3 | - | - |
| 294 | 03837714 | M | 3year | 0.0 | 0.8 | - | - |
| 295 | 03979600 | M | 6year | 0.5 | 0.3 | + | - |
| 296 | 02879095 | M | 6year | 0.2 | 0.5 | - | - |
| 297 | 90213983 | M | 3year | 0.0 | 0.8 | - | - |
| 298 | 03582070 | W | 4year | 0.0 | 0.2 | - | - |
| 299 | 90104638 | W | 4year | 0.1 | 0.6 | - | - |
| 300 | 90081117 | W | 4year | 0.2 | 0.7 | - | - |
