# Supplementary figures and images for: Establishment and Performance Evaluation of Multiplex PCR-Dipstick DNA Chromatography for Mycoplasma pneumoniae and Chlamydia pneumoniae Rapid Detection
Source: Can J Infect Dis Med Microbiol. 2023 Sep 28;2023:6654504. doi: 10.1155/2023/6654504 (PMC10555492; doi:10.1155/2023/6654504)

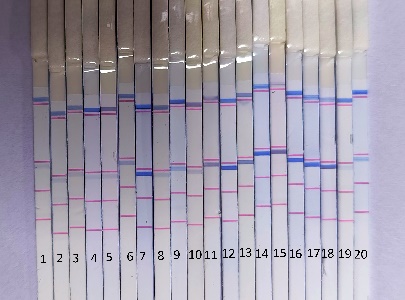

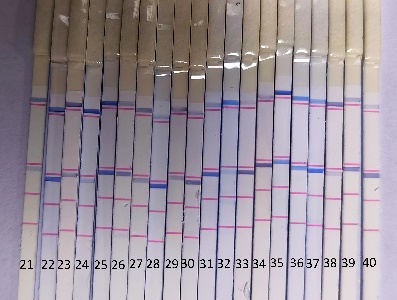

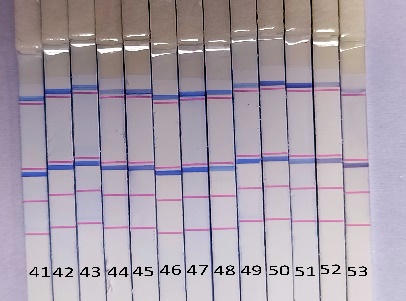


53cases positive for MP


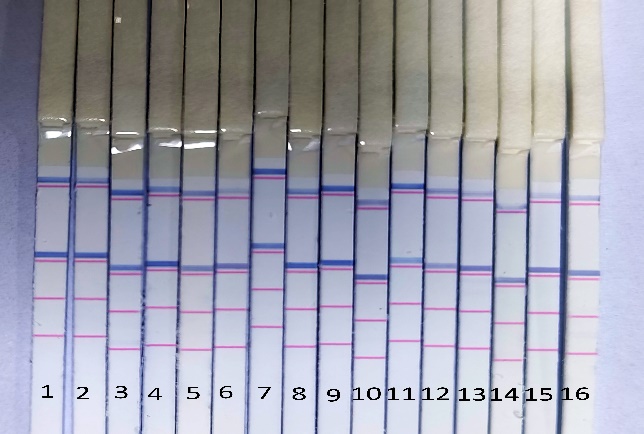

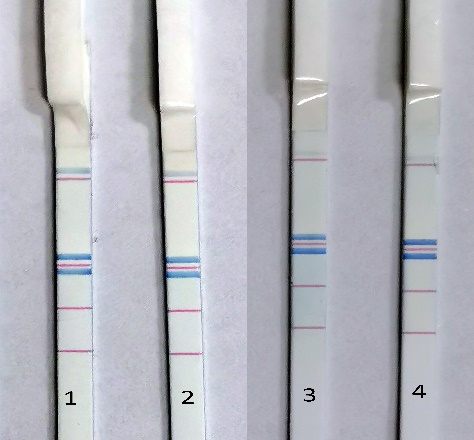


16 cases positive for CP 4 cases simultaneously positive for MP and CP

Supplement: Supplementary Materials — We have uploaded a Word file named “Clinical Sample Test Results” to Supplementary Materials, which contain MP-IgM and CP-IgM S/CO values and corresponding multiplex PCR-dipstick DNA chromatography results. Five images were uploaded to Supplementary Materials, which contain the blue intensity of 53cases positive for MP, 16 cases positive for CP, and 4 cases simultaneously positive for MP and CP by the multiplex PCR-dipstick chromatography assay. [file 6654504.f1.zip › 5 images .docx]
